# *Echinococcus ortleppi* Infections in Humans and Cattle, France

**DOI:** 10.3201/eid2012.140641

**Published:** 2014-12

**Authors:** Frédéric Grenouillet, Gérald Umhang, Francine Arbez-Gindre, Georges Mantion, Eric Delabrousse, Laurence Millon, Franck Boué

**Affiliations:** World Health Organization Collaborating Center for Prevention and Treatment of Human Echinococcosis and French National Reference Center on Alveolar Echinococcosis, Besançon, France (F. Grenouillet, F. Arbez-Gindre, G. Mantion, E. Delabrousse, L. Millon);; Chrono-Environnement Unité Mixte de Recherche 6249, Centre National de la Recherche Scientifique et Université de Franche-Comté, Besançon (F. Grenouillet, L. Millon);; Agence Nationale de Sécurité Sanitaire de l'Alimentation National Reference Laboratory for *Echinococcus* spp., Malzéville, France (G. Umhang, F. Boué)

**Keywords:** Echinococcus ortleppi, parasites, France, humans, cattle, infection, tapeworms

## Abstract

In 2011 and 2012, liver infections caused by *Echinococcus ortleppi* tapeworms were diagnosed in 2 humans in France. In 2012, a nationwide slaughterhouse survey identified 7 *E. ortleppi* infections in cattle. The foci for these infections were spatially distinct. The prevalence of *E. ortleppi* infections in France may be underestimated.

Cystic echinococcosis (CE) is a zoonotic disease caused by the taeniid tapeworm *Echinococcus granulosus* sensu lato (*1*). This neglected disease is distributed worldwide and causes illnesses in humans and animals (*2*). During the past few decades, the taxonomic status of *E. granulosus* has been uncertain; varying classifications have been proposed for its species, subspecies, and genotypes. This taxon is now recognized as a complex of at least 5 distinct species that encompass 10 genotypes with different host specificities: *E. granulosus* sensu stricto (genotypes G1–G3), *E. equinus* (G4), *E. ortleppi* (G5), and *E. canadensis* (G6–G10) (*3*). *E. ortleppi*, first described in South Africa (*4*,*5*), has a dog/cattle life cycle and is reported to have low pathogenicity for humans. This species is prevalent in South America but has been reported only sporadically on other continents (*6*,*7*). 

In France, the annual incidence rate of CE was stable during 2005–2012 at ≈0.18 cases per 100,000 inhabitants, or 110 new cases per year (D. Van Cauteren, unpub. data). A nationwide slaughterhouse survey in 1989 revealed an average infection rate of 0.13% in cattle and 0.42% in sheep and goats (*8*). More recently, low prevalence in slaughterhouses was described in southern France (3 and 4 cases per 100,000 in cattle and sheep, respectively), and only *E. granulosus* sensu stricto larval tapeworms were identified (*9*). We provide evidence of infection with *E. ortleppi* larval tapeworms in France in humans and in cattle and delineate 4 distinct spatial localizations for these infections.

## The Cases

**Figure 1:**
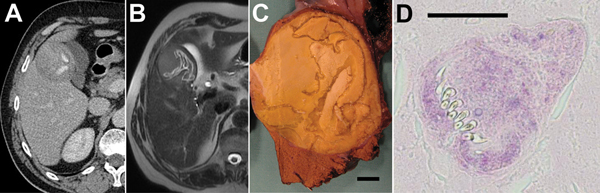
Results of testing in a 63-year-old man from the Jura *département* (eastern France), who was diagnosed with infection with *Echinococcus ortleppi* larval tapeworms in 2001. A) Abdominal computed tomography scan; B) magnetic resonance imaging; and C) macroscopic morphologic examination of operative specimen. All show lesions with a detached endocyst and calcified matrix; scale bar in panel C indicates 1 cm. D) Microscopic examination shows evidence of protoscoleces in the matrix (hematoxylin and eosin stain; scale bar indicates 50 µm).

In 2011, a 63-year-old man from the Jura *département* (eastern France) sought treatment for moderate pain in the right hypochondrium. Ultrasound examination revealed 2 hyperechoic liver nodules (6 and 3 cm in diameter) in segment V. Computed tomography (CT) scan of the abdomen showed atypical lesions, suggesting a tumor, but magnetic resonance imaging revealed well-defined cysts with internal structure suggestive of CE, with detached endocysts (Figure 1, panels A, B). CE had initially been ruled out because of a negative result from serologic testing for *Echinococcus* (ELISA using *E. granulosus* vesicular fluid). A right hepatectomy was performed, and histopathologic examination of the operative specimen revealed many protoscoleces and detached layers (Figure 1, panels C, D), confirming CE. Retrospective re-analysis of the patient’s serum sample showed evidence of antibodies against *Echinococcus*, demonstrated by *E. granulosus* hemagglutination (Fumouze, Levallois, France) at 1:80 and by Western blot (LDBio Products, Lyon, France) showing the typical 7-kDa band. The patient’s medical history indicated that he had probably been infected ≈10 years previously through contact with his son’s dog in a cattle-breeding, middle-altitude mountainous region (the Haute-Savoie *département* in the French Alps). Ultrasound and serologic screening excluded other CE cases in the patient’s family.

In 2012, a 39-year-old woman in the Vendée *département* (western France) sought treatment for abdominal pain and fever; testing showed a typical 7-cm diameter, well-defined liver cyst in segment VII, pathognomonic of CE, despite negative results for *Echinococcus* on serologic testing (ELISA for *E. granulosus*, e.g., hemagglutination Fumouze, Western blot, LDBio). Pericystectomy and cholecystectomy were performed. Histopathologic examination of the liver cyst revealed calcified content with protoscoleces. The patient had been living near farms for >20 years in an area with many stray dogs and where livestock carcasses are not disposed of quickly. Ultrasound screening excluded CE in her husband and children.

**Figure 2:**
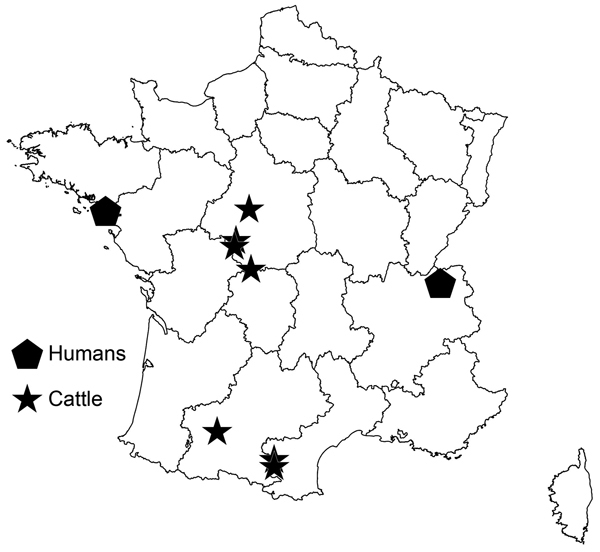
Geographic locations of human and cattle cases of *Echinococcus ortleppi* infection, France, 2011–2012.

During 2012, a nationwide slaughterhouse survey for CE was conducted in France identified *E. ortleppi* infections in 7 cattle. During meat inspection, cysts were systematically sampled, then stored for genotyping. Molecular methods identified *E. ortleppi* tapeworms in 7 cattle that had lung cysts. All cases except 1 showed fertile cysts with numerous protoscoleces. Mean age of the animals was 10 (range 3–14) years. The cattle were of 4 breeds; 6 were meat animals, and 1 was a dairy animal. All 7 animals came from 2 foci, 1 in central France (n = 4) and 1 in southwestern France (n = 3); the diameters of these foci were 130 and 160 km, respectively (Figure 2). None of the animals originated from the same herd, but in each focus area, 2 animals came from herds <10 km apart. Three animals had lived on another farm, but in each case, the farm was in the same focus area.

We conducted molecular analyses of fresh cyst tissue samples from the 2 patients and the 7 cattle using PCR-based DNA sequencing of 2 genes, the mitochondrial cytochrome *c* oxidase 1 (*cox1*) and ATPase subunit 6 (*ATP6*), as previously described (*10*,*11*). Samples from all cattle and the female patient showed the same sequences of the *cox1* gene (GenBank accession no. KC430087) and 100% identity with reference *E. ortleppi* gene sequences available in the GenBank database, determined by using BLAST software (http://www.ncbi.nlm.nih.gov/blast). The sample from the male patient, however, had a different *cox1* sequence (GenBank accession no. KJ624625), with 1 substitution. All cattle and human lesions had the same *ATP6* sequence (GenBank accession no. KC430091), showing 100% identity with a reference sequence from GenBank (accession no. DQ318953). All findings were consistent with *E. ortleppi* infection.

## Conclusions

Infection with *E. ortleppi* larval tapeworms is rarely diagnosed in humans; a literature search found only 8 reported cases worldwide (*7*). Since 1984, in Europe, infections have been reported in 1 human in the Netherlands (*12*) and in 1 bovid from Italy (*13*). The 2 foci of cattle infections we found did not spatially correspond to the human cases or to the major cattle foci of CE previously identified in France (*8*). Close proximity to cattle and dogs suggests autochthonous infection for the human cases. For the suspected infection area of the male patient (Haute-Savoie), only *E. granulosus* sensu stricto infection had previously been reported in cattle (*9*).

False-negative serologic results are frequent in patients with CE; 10%–20% of patients with hepatic cysts, especially old and devolving lesions, do not produce detectable specific serum antibodies (*1*). Moreover, antigens used for serologic tests are manufactured from *E. granulosus* sensu stricto G1, which may be antigenically different from *E. ortleppi*. The lack of immunoreactivity in patients with *E. ortleppi* infection has led, and may lead, to underdiagnosis.

*E. ortleppi* tapeworms are called the “Swiss cattle strain,” but some authors have suggested that this species may become extinct in Europe as a result of fewer opportunities for transmission between cattle and dogs (*14*,*15*). However, the fertile lesions observed in cattle and humans in our study may attest to the ability of the parasite to adapt to its hosts. Because home slaughter and deaths of animals without appropriate carcass disposal are rare events in Western European countries, this fertility may compensate for the decreased probability of interactions between cattle-infected viscera and dogs. Because molecular data are scarce, it is difficult to determine how widespread *E. ortleppi* tapeworms are in France or to evaluate the potential for human infection with this parasite.

In summary, our findings of *E. ortleppi* larval tapeworm infections in France highlight the need to enhance national surveillance efforts. *E. ortleppi* tapeworms found in cases of human CE should be systematically genotyped to determine patterns of infection, and meat from slaughterhouses in the 4 identified foci should be inspected carefully. In addition, information on dog infection status and livestock production practices at local cattle farms should be compiled to help identify anomalies in the regulated procedures for carcass treatment and disposal that help maintain the *E. ortleppi* tapeworm life cycle.
